# Lymphoma Mimicking Pyoderma Gangrenosum: A Case Report

**DOI:** 10.7759/cureus.63970

**Published:** 2024-07-06

**Authors:** Esra Nurlu Temel, Fusun Z Akcam, Ahmet Ozdemir, Ayse Hilal Turker, Raşit Akdeniz, Gul Ruhsar Yilmaz

**Affiliations:** 1 Department of Infectious Diseases, Suleyman Demirel University, Isparta, TUR; 2 Department of Pathology, Suleyman Demirel University, Isparta, TUR

**Keywords:** pyoderma gangrenosum, rheumatoid arthritis, cellulite, high-grade b-cell lymphoma, necrotizing soft tissue infection (nsti)

## Abstract

Some inflammatory conditions, such as pyoderma gangrenosum, and tumoral conditions, such as lymphoma, may appear as soft tissue infections. Herein, a cutaneous lymphoma patient who was hospitalized with a diagnosis of soft tissue infection and was considered to have pyoderma gangrenosum during follow-up is presented. Immediate histopathological examination should be recommended to diagnose skin soft tissue lesions, especially long-term and unresponsive to treatment.

## Introduction

Pyoderma gangrenosum is a rare inflammatory skin disease characterized by the formation of painful pustules or nodules that gradually enlarge into ulcers. The lesions that initially manifest as painful nodules or pustules subsequently evolve into ulcers with elevated edges that are tender to the touch and tend to enlarge gradually [1]. The disease may be observed in association with systemic diseases, including inflammatory bowel diseases, rheumatoid arthritis, and polyclonal gammopathy, or may develop without any underlying disease. In the ulcer stage, lesions may appear similar to abscesses and infected skin ulcers [2]. Furthermore, the skin may also show a proclivity toward malignant transformation, with the lymphatic tissue formed by the aforementioned defense cells, as with other lymphoid tissues within the body [3]. Primary cutaneous lymphomas are neoplastic proliferation of lymphocytes in the skin. These are the second most common form of non-Hodgkin lymphoma. Primary cutaneous B-cell lymphoma is defined as B-cell lymphomas present in the skin without any extracutaneous involvement at the time of diagnosis. Cutaneous B-cell lymphomas comprise approximately 25% of all cutaneous lymphomas. They are divided into three main morphological groups: primary cutaneous marginal zone lymphoma, cutaneous follicle-center lymphoma, and diffuse large B-cell lymphoma, leg type [4].

Herein, a cutaneous lymphoma patient who was hospitalized with a diagnosis of soft tissue infection and was considered to have pyoderma gangrenosum during follow-up is presented.

## Case presentation

**Figure 1:**
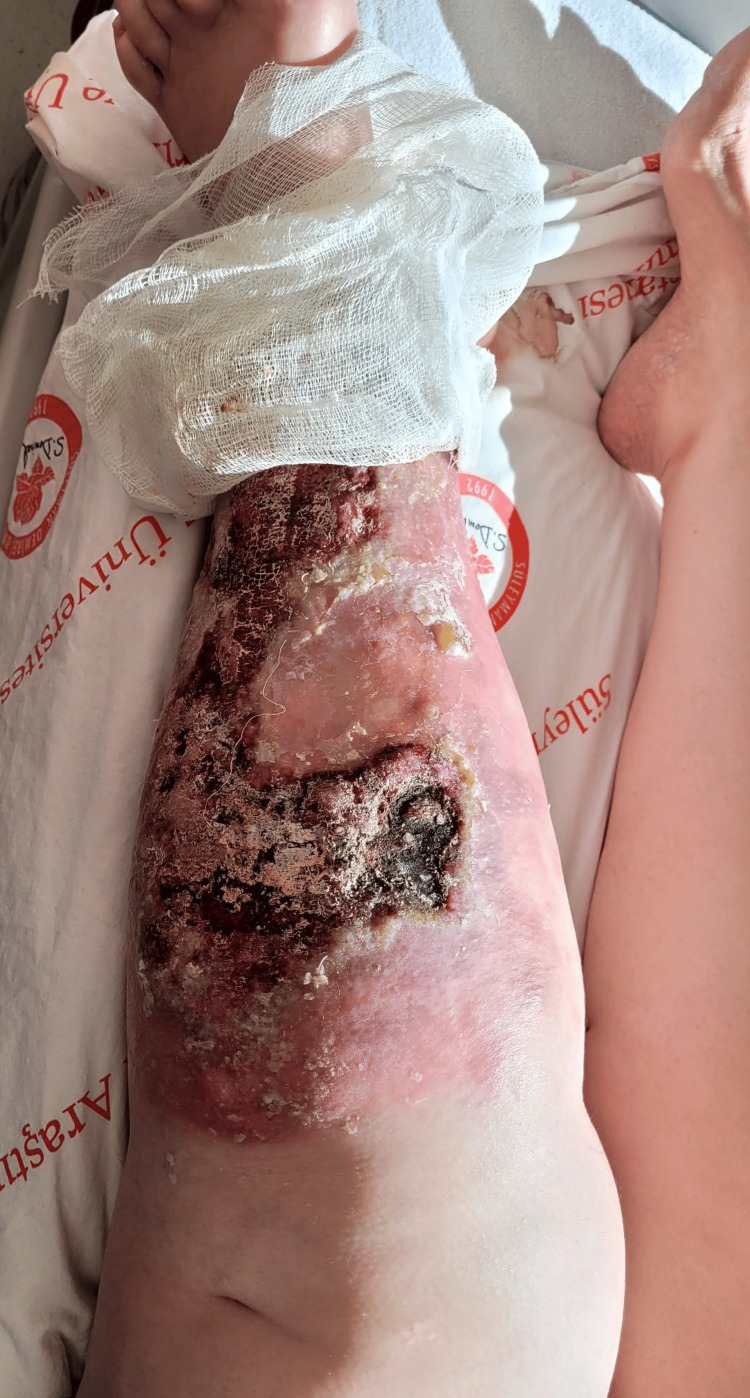
The lesion, when she presented to our clinic, accompanied by necrotic, hemorrhagic dry discharge on an erythematous pigmented base extending from below the left knee to the distal tibia

**Figure 2:**
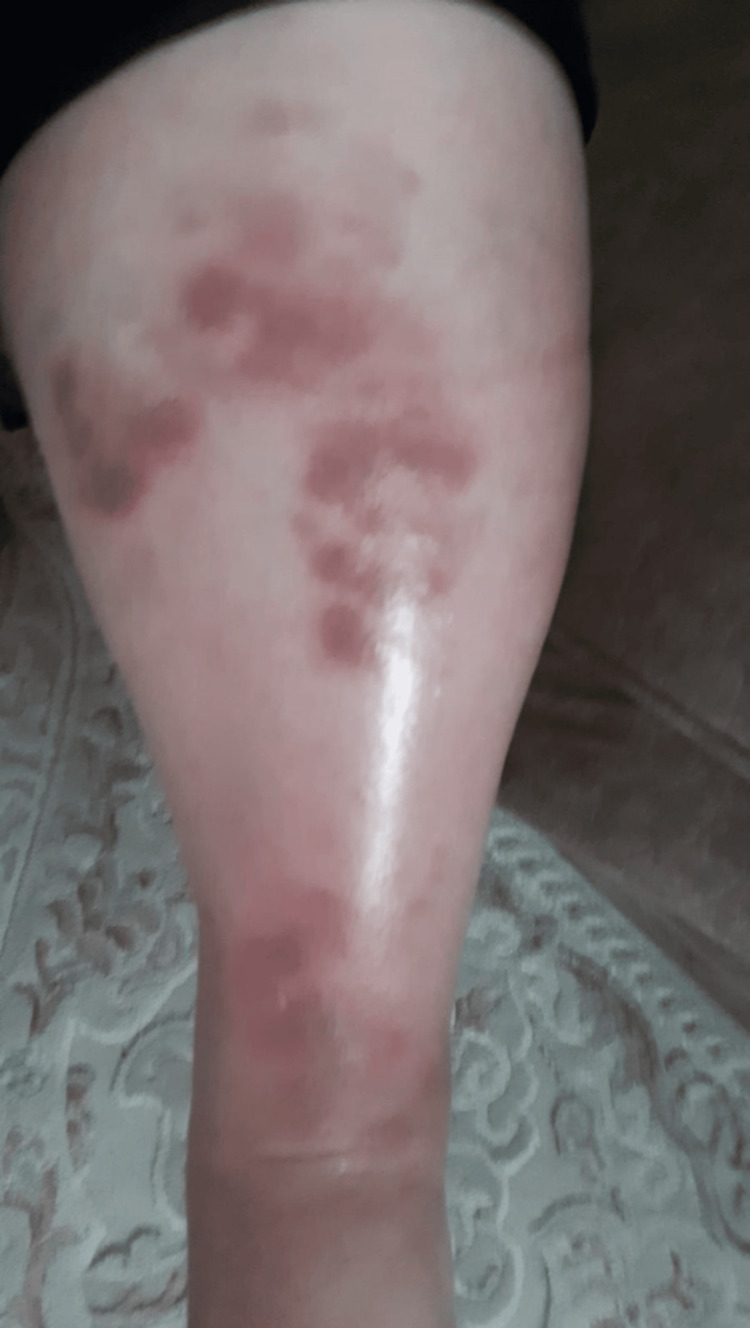
Initial symptoms manifested as swelling in the left leg and the development of small purple lesions on the anterior surface of the leg approximately three months before

**Figure 3:**
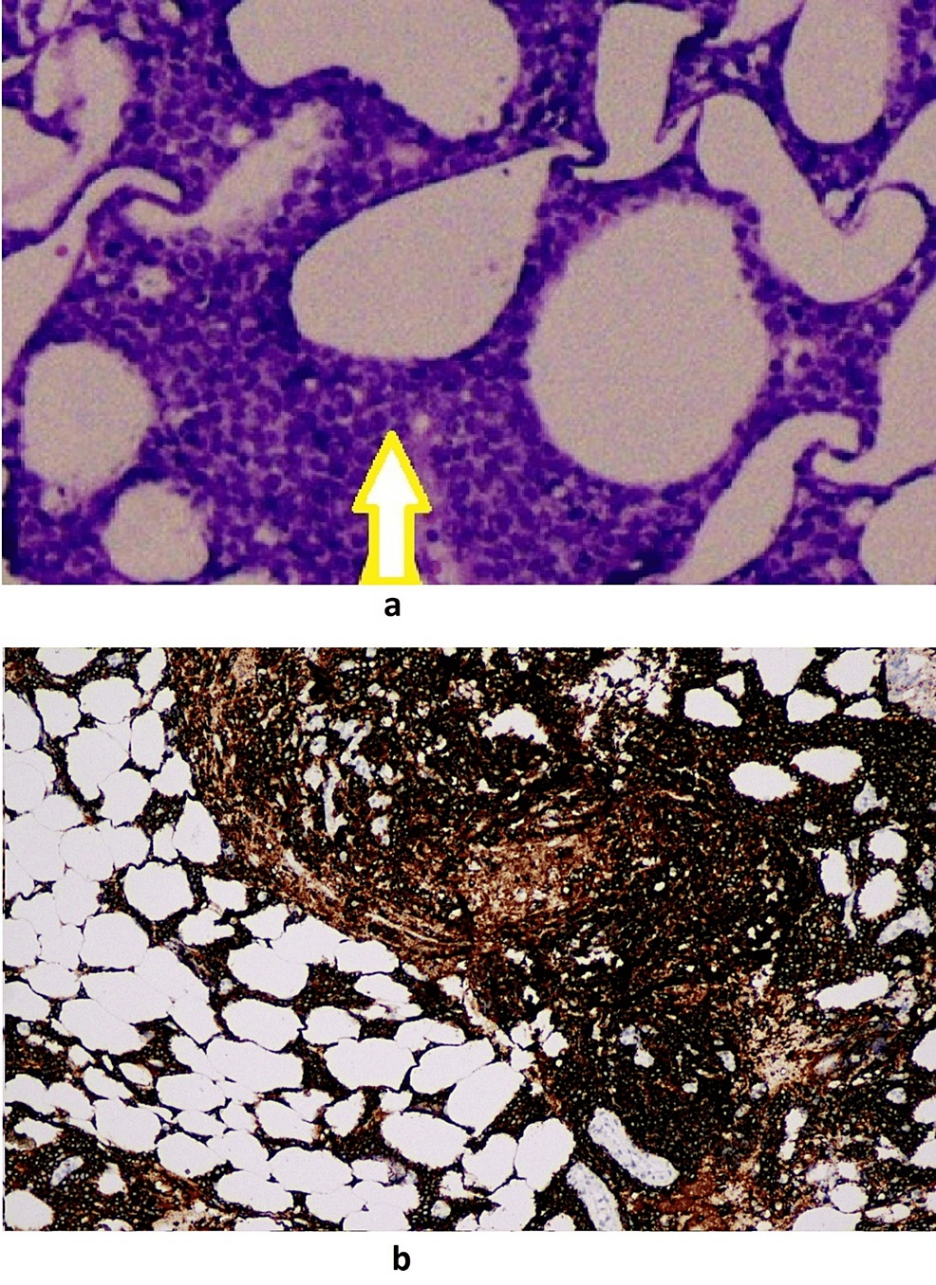
(a) Atypical lymphocytes infiltrating adipocytes (20x H&E); (b) CD20 expressing atypical lymphocytes that infiltrate adipocytes (DAB x100)

A 59-year-old woman with rheumatoid arthritis presented to our clinic with a complaint of a painful wound on the left leg. On physical examination, a lesion extending from below the left knee to the distal tibia was observed on an erythematous pigmented ground accompanied by necrotic, hemorrhagic dry discharge. Furthermore, there was a notable increase in the patient's leg temperature and diffuse edema (Figure 1). The patient, believed to have a soft tissue infection, was admitted to the hospital for intravenous antibacterial treatment. Tissue and blood cultures were obtained. Laboratory tests revealed a CRP level of 173 mg/L, a procalcitonin level of 0.21 ng/mL, a WBC count of 11,200 uL (with 86% neutrophils and 6% lymphocytes), and the initiation of ampicillin-sulbactam at a dose of 4x2 g and ciprofloxacin at a dose of 2x500 mg. It was established from the patient's medical history that he was undergoing treatment for rheumatoid arthritis. Her initial symptoms manifested as swelling in the left leg and the development of small purple lesions on the anterior surface of the leg approximately three months before his presentation to our facility (Figure 2). Due to the lack of regression in the lesion on the fourth day of treatment and the growth of MR-CNS in the tissue culture taken during the patient's hospitalization, ampicillin sulbactam was discontinued, and teicoplanin was administered instead. A biopsy of the skin and soft tissues was performed, with a preliminary diagnosis of pyoderma gangrenosum. The patient was then commenced on a course of prednisolone at a dosage of 60 mg per day. The skin biopsy yielded inconclusive results but was consistent with a diagnosis of pyoderma gangrenosum. Histopathologically, there were findings of ulcer, abscess, granulation tissue, panniculitis, and fat necrosis in the epidermis. The anticipated clinical response to steroid treatment was not observed. The result of the soft tissue biopsy was reported as high-grade B-cell lymphoma (Figure 3). The antimicrobial and steroid treatment was discontinued, and the patient was referred to the hematology clinic.

## Discussion

A multitude of factors may cause ulcerated skin lesions. In addition to skin-soft tissue infections, ulcerated skin lesions may be observed in venous or arterial vasculitis cases, primary or metastatic tumoral formations, and skin inflammatory diseases, such as pyoderma gangrenosum. The differential diagnosis is typically based on the clinical findings accompanying the lesion and the response to treatment. To reach a definitive diagnosis, histological examination is often necessary. 

It has been reported that approximately 50% of patients with pyoderma gangrenosum have an underlying systemic disease [5]. These include inflammatory bowel diseases, arthritis, leukemia, hepatitis, primary biliary cirrhosis, malignancy, and monoclonal gammopathies [2,5]. The patient had been receiving methotrexate for 20 years, with a diagnosis of rheumatoid arthritis. T-cell lymphoma ulcers can initially be misdiagnosed as pyoderma gangrenosum due to ulceration secondary to massive epidermotropism and a scarcity of atypical cells. In the literature, cutaneous T-cell lymphomas have been reported to be confused with pyoderma gangrenosum [6,7]. In this case, the inflammatory findings observed in the skin biopsy sample were consistent with the clinical prediagnosis of pyoderma gangrenosum. However, the diagnosis was confirmed as lymphoma following pathological examination of deep soft tissue.

Primary cutaneous lymphomas are a heterogeneous group that includes cutaneous T-cell and B-cell lymphomas. When diagnosed, they are mostly localized to the skin. Approximately 80% is T-cell lymphoma and 20% is B-cell lymphoma. For diagnosis, clinical, histopathology, immunotyping, and gene analyses should be evaluated together [8]. Primary cutaneous marginal zone lymphoma, primary cutaneous follicle center cell lymphoma, and primary cutaneous diffuse large cell lymphoma are the main B-cell cutaneous lymphomas. Primary cutaneous B-cell lymphoma, leg type, is mainly a disease of elderly women. The median age at diagnosis is 70 years. Clinical presentation includes red or livid-red tumors on one or both legs. Extracutaneous lesions are often seen. The histopathological evaluation shows monomorphic, diffuse infiltration of the dermis, with no involvement of the epidermis. Staging of patients diagnosed with primary cutaneous lymphoma is important for their follow-up, prognosis, and treatment [9].

In treating pyoderma gangrenosum, systemic corticosteroids may be administered as a monotherapy or combined with salazopyrin and other immunosuppressive agents. In some patients, high-dose steroid treatment of 100-200 mg/day may be required [10]. A biopsy was performed on the patient due to the initial appearance of the wound (Figure 1) and the lack of response to antibacterial treatment. Steroid treatment was initiated without awaiting the biopsy result. It is acknowledged that there is no definitive treatment approach for pyoderma gangrenosum and that treatment response is unpredictable [11]. So, despite the absence of improvement in the lesion following the commencement of steroid treatment, no modification in the steroid treatment regimen was contemplated until the soft tissue biopsy results were available.

## Conclusions

In conclusion, it is recommended that ulcerative skin lesions be promptly subjected to pathological investigation, particularly when accompanied by pain and tenderness of long duration and an inadequate response to treatment. An accurate diagnosis can prevent the administration of an insufficient treatment regimen, which can result in significant complications for these patients.

## References

[1] Callen JP (1998). Pyoderma gangrenosum. Lancet.

[2] Wollina U, Haroske G (2011). Pyoderma gangraenosum. Curr Opin Rheumatol.

[3] Akcam FZ, Unal O, Kaya O, Erkilic G, Yilmaz GR, Baspinar S (2020). Lymphoma erysipeloides. IDCases.

[4] Sokołowska-Wojdyło M, Olek-Hrab K, Ruckemann-Dziurdzińska K (2015). Primary cutaneous lymphomas: diagnosis and treatment. Postepy Dermatol Alergol.

[5] Powell FC, Su WP, Perry HO (1996). Pyoderma gangrenosum: classification and management. J Am Acad Dermatol.

[6] Flora A, Gibson M, Toon C, Rawson R, Lowe P (2021). Primary cutaneous CD8+ aggressive epidermotropic T-cell lymphoma mimicking pyoderma gangrenosum. Indian J Dermatol Venereol Leprol.

[7] Ilter N, Keskin N, Adisen E, Erdem O, Ogut B (2021). Primary cutaneous aggressive epidermotropic CD8(+) T cell lymphoma mimicking pyoderma gangrenosum. Australas J Dermatol.

[8] Gilson D, Whittaker SJ, Child FJ (2019). British Association of Dermatologists and U.K. Cutaneous Lymphoma Group Guidelines for the management of primary cutaneous lymphomas 2018. Br J Dermatol.

[9] Sutton AM, Hurley MY (2015). Clinical practice guidelines for cutaneous lymphomas. Mo Med.

[10] Futami H, Kodaira M, Furuta T, Hanai H, Kaneko E (1998). Pyoderma gangrenosum complicating ulcerative colitis: successful treatment with methylprednisolone pulse therapy and cyclosporine. J Gastroenterol.

[11] Miller J, Yentzer BA, Clark A, Jorizzo JL, Feldman SR (2010). Pyoderma gangrenosum: a review and update on new therapies. J Am Acad Dermatol.

